# The Blue-Green Sensory Rhodopsin SRM from *Haloarcula marismortui* Attenuates Both Phototactic Responses Mediated by Sensory Rhodopsin I and II in *Halobacterium salinarum*

**DOI:** 10.1038/s41598-019-42193-y

**Published:** 2019-04-05

**Authors:** Jheng-Liang Chen, Yu-Cheng Lin, Hsu-Yuan Fu, Chii-Shen Yang

**Affiliations:** Department of Biochemical Science and Technology, National Taiwan University, Taipei, 10616 Taiwan

## Abstract

Haloarchaea utilize various microbial rhodopsins to harvest light energy or to mediate phototaxis in search of optimal environmental niches. To date, only the red light-sensing sensory rhodopsin I (SRI) and the blue light-sensing sensory rhodopsin II (SRII) have been shown to mediate positive and negative phototaxis, respectively. In this work, we demonstrated that a blue-green light-sensing (504 nm) sensory rhodopsin from *Haloarcula marismortui*, SRM, attenuated both positive and negative phototaxis through its sensing region. The *H. marismortui* genome encodes three sensory rhodopsins: SRI, SRII and SRM. Using spectroscopic assays, we first demonstrated the interaction between SRM and its cognate transducer, HtrM. We then transformed an SRM-HtrM fusion protein into *Halobacterium salinarum*, which contains only SRI and SRII, and observed that SRM-HtrM fusion protein decreased both positive and negative phototaxis of *H. salinarum*. Together, our results suggested a novel phototaxis signalling system in *H. marismortui* comprised of three sensory rhodopsins in which the phototactic response of SRI and SRII were attenuated by SRM.

## Introduction

Solar light has been the main energy source propelling the biosphere since early Earth. Many organisms, including archaea, bacteria, protists and plants^1–3^, evolved specific photosynthetic, phototropic or photosensing systems^4^ driven by light of the visible or near-infrared spectra^5^ for energy capture or physiological responses.

In halophilic archaea, four major types of microbial rhodopsins have been identified. First, bacteriorhodopsin is a light-driven outward proton pump triggered by 550 nm light that partners with the F1Fo ATP synthase to harvest solar energy. Second, halorhodopsin, a light-driven inward chloride pump activated by 580 nm light, is to maintain cellular osmotic pressure under extreme salinity^6^. In addition, we recently reported that halorhodopsin pumps chloride through a proton partnership^7^ and thus, its physiological role requires more investigation. The third and fourth types of rhodopsins are the red light-sensing (585 nm) sensory rhodopsin I (SRI) and the blue light-sensing (485 nm) sensory rhodopsin II (SRII), which mediate photo-attractant and photorepellent responses, respectively^8–10^. The combination of attractant and repellent responses localize microbes to an optimal habitat for capturing light energy, while preventing effects of harmful light of shorter wavelengths.

Sensory rhodopsin regulates phototactic signalling proteins by interacting with a specific cognate partner transducer called the halobacterial transducer of rhodopsin, or Htr^11,12^. The structure of the transducer from the N- to C-terminus includes two transmembrane domains, a HAMP (Histidine kinases, Adenylate cyclases, Methyl accepting proteins and Phosphatases) domain, a methyl-accepting domain and a CheA/CheW baseplate that combines the six tips of the transducer into a trimer of dimer^13^. The methyl-accepting sites can be regulated through demethylation by CheB as well as auto-methylation by CheR, and the methylation status affects the flexible states of the transducer^14^. Thus, SR-Htr complex transmits the signal from the light activated SR to the downstream regulation of flagella apparatus^15^. Phototactic signalling pathways were previously shown in both the red-blue (RB) sensory system of *Halobacterium salinarum*^16^ and the blue light only sensory system of *Natronomonas pharaonis*^14^. Positive phototaxis is triggered through red light absorption by SRI-HtrI complex. At the same time, negative phototaxis is also able to be triggered through the two-photon pathway of the SRI-HtrI photocycle^17^. SRII-HtrII absorbs the blue spectrum and induces negative phototactic signalling. Nonetheless, SRI-HtrI and BR are absent at the same time in blue only sensory system*, N. pharaonis*^18^. Therefore, the two sensory rhodopsins from *H. salinarum*, SRI and SRII, have been typically used as a model system to study phototaxis^16,19,20^. SRI and SRII, together with their transducers HtrI and HtrII, mediate positive and negative phototaxis, respectively. The distribution of these microbial rhodopsins implies that positive phototaxis mediated by SRI-HtrI localizes haloarchaea to an optimal environment for BR to exert its light-driven outward proton pumping, while the function of negative phototaxis regulated by SRII-HtrII is to avoid photooxidative damage when light is not the primary energy source in some halophilic archaea^21^. Taken together, the physiological role among microbial rhodopsins should be further investigated because the distribution of microbial rhodopsins varies in different haloarchaea based on their genomes^22,23^.

In 2010, we reported a unique six-rhodopsin system in *Haloarcula marismortui*^24^ comprised of two bacteriorhodopsins, one halorhodopsin and three sensory rhodopsins^24,25^. Two of the sensory rhodopsins are homologous to *H. salinarum* SRI and SRII. We named the third sensory rhodopsin “SRM”, designating it as the middle-type between SRI and SRII. In this three-sensory rhodopsin system, SRI and SRII were shown to regulate positive and negative phototaxis, respectively^8–10^. On the other hand, SRM responded to blue-green light at 504 nm^24,25^, a wavelength situated between those sensed by SRI (585 nm) and SRII (468 nm). Immediately downstream of the SRM gene, there is an open reading frame encoding a cognate partner transducer, HtrM^24^, which intriguingly lacks the histidine kinase domain universally present in all transducers^26^.

In this work, we investigated the physiological function of SRM-HtrM. We first showed the interaction between SRM and HtrM by *in vitro* spectroscopic assays. Meanwhile, we developed a microscopy-based method to quantify both positive and negative phototaxis in *H. marismortui* and *H. salinarum*. *H. marismortui* SRM-HtrM fusion protein was then transformed into *H. salinarum*, which contains its endogenous SRI/SRII sensory rhodopsin system. Phototactic measurements showed that SRM-HtrM attenuated phototactic responses to green light mediated by both SRI and SRII in *H. salinarum*.

## Results

**Figure 1:**
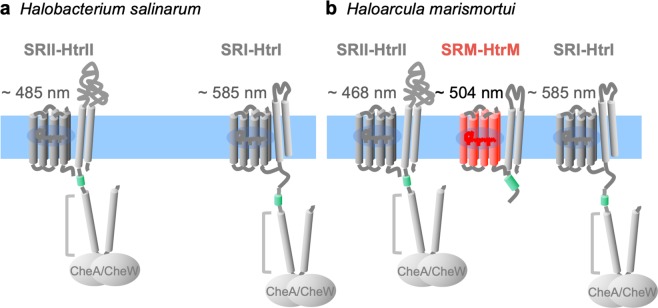
Sensory rhodopsin systems in *H. salinarum* and *H. marismortui*. Schematic of sensory rhodopsins (SRI, SRII and SRM) and their cognate transducers (HtrI, HtrII and HtrM) in *H. salinarum* (**a**) and *H. marismortui* (**b**). The maximum absorbances of purified sensory rhodopsins are shown above the drawings. Short light green cylinders indicate the cytoplasmic HAMP domains of the cognate transducers. Long grey cylinders indicate the methyl-accepting domains. The cytoplasmic tips of HtrI and HtrII are proposed as the interaction sites for CheA and CheW. Note that HtrM lacks the cytoplasmic domains required for CheA and CheW interactions.

SRM-HtrM is a member of the three-sensory rhodopsin system in *H. marismortui* (Fig. 1b)^24^. Among them, HmSRI responses optimally at 585 nm to mediate positive phototaxis, while HmSRII is maximally excited by 468 nm to trigger negative phototaxis. SRM serves as the third sensory rhodopsin which absorbs maximally at 504 nm, with HtrM functioning as its cognate transducer based on both genomic analysis and indirect interaction assays^24^. For maximal absorption, the activation wavelength of SRM-HtrM fusion protein was found to be at 504 nm over the UV-Vis spectrum; this wavelength sits between those of HsSRI and HsSRII (Fig. 1a). The generally accepted model for phototactic signalling is that chemotactic proteins (e.g., CheB and CheR) engage in the methylation of transducers and in histidine kinase activities to regulate flagella. Intriguingly, HtrM lacks both a methyl-accepting domain and the cytoplasmic tip for CheA/CheW interactions (Fig. 1b). We were intrigued by the sole HAMP domain present on the cytoplasmic side of HtrM and therefore investigated the interaction between SRM and HtrM and their potential roles in phototactic signalling.

### ***In vitro*** interaction of SRM and HtrM

A proper interaction between SRM and HtrM is the first step in the signalling cascade from the light-activated SRM to the flagella. To thoroughly examine the interaction between SRM and HtrM, we used three independent assays to evaluate the interaction among the purified SRM-HtrM fusion complex.

**Figure 2:**
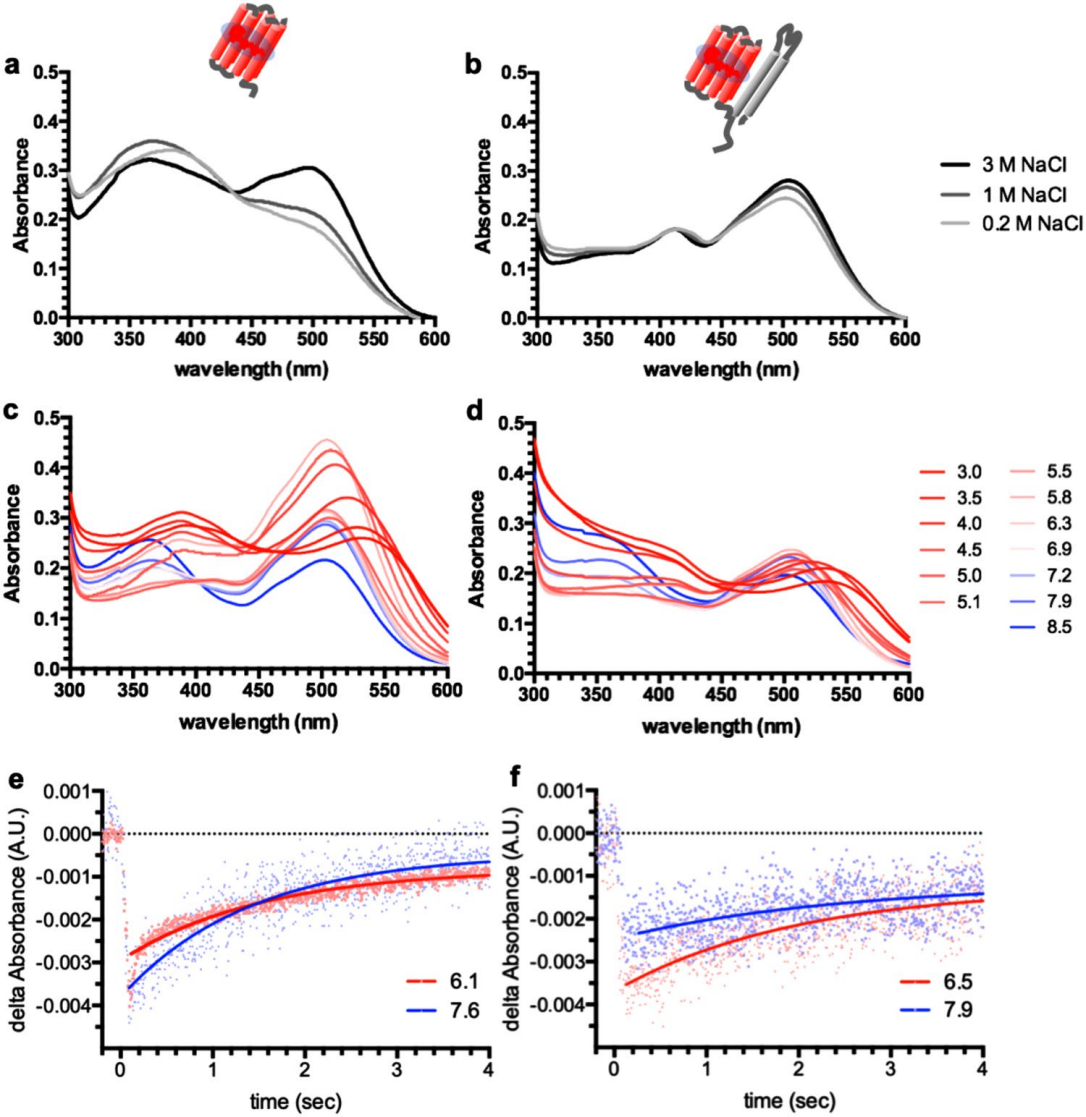
Interaction of detergent-solubilized SRM and HtrM. As indicated by the schematics at the top, the left (**a**,**c**,**e**) and right (**b**,**d**,**f**) columns show the results of SRM alone and the SRM-HtrM fusion proteins, respectively. (**a**,**b**) Absorbance spectra under different salt concentrations with a buffering system of 50 mM MES, pH 5.8, 0.02% DDM. (**c**,**d**) Absorbance spectra under different pH conditions (as indicated by numbers) and in 4 M NaCl, 0.02% DDM. Different pH buffering systems (50 mM) were adopted as follow: pH 3.0–5.5: sodium citrate; pH 5.83–6.35: MES; pH 6.9–7.2: MOPS; pH 7.9–8.5: Tris-HCl. All absorbance spectra were normalized so that absorbance at 280 nm = 1.0. (**e**,**f**) Photocycle measurements of detergent-solubilized SRM and SRM-HtrM under acidic (4 M NaCl, 0.02% DDM, 50 mM MES) and alkaline (Tris) conditions. The kinetic (i.e., Tau values) of all photocycle plots is listed in Table 1.

First, we found that SRM was more optically stable in the presence of HtrM under varying salt concentrations. Sensory rhodopsins from other organisms were shown to exhibit higher absorbance stability and photocycle kinetics in the presence of their cognate transducers under different salt concentrations^27^. A UV-Vis spectrum scan was carried out for both SRM alone and SRM-HtrM fusion protein. Both SRM and SRM-HtrM fusion protein were expressed and purified from *E. coli* and then suspended in different salt concentrations (200 mM, 1 M and 3 M NaCl) (Fig. 2a,b). The UV-Vis spectrum of SRM-HtrM fusion was markedly more consistent in different salt concentrations (Fig. 2b) compared to the spectrum of SRM alone (Fig. 2a). The absorbance profile of SRM alone showed an approximate 30% decrease by 504 nm, which was accompanied by an increase by 400 nm (Fig. 2a), indicating the accumulation of proteins trapped in intermediate states^28,29^. These results suggested that the interaction between SRM and HtrM stabilized the optical characteristics of SRM.

Second, we observed that SRM-HtrM fusion protein was more optically stable than SRM alone under extreme pH conditions ranging from 3.0 to 8.5 (Fig. 2c,d). Under pH conditions equal to and above 4.0, SRM-HtrM fusion protein had a stable spectrum profile without a significant shift in the maximum absorbance at 504 nm (Fig. 2d). When the pH was lower than 4.0, a shift in the maximum absorbance towards red light started to occur and was accompanied by an increase in the 410 nm fraction. On the other hand, the samples with SRM alone had high oscillations in their UV-Vis scanning profiles when the pH was equal to and above 4.0, with the absorbance by 504 nm decreasing with increasing pH up to 8.5. Such observations hinted a physical interaction between SRM and HtrM.

Third, we found that in the presence of HtrM, the photocycle kinetics of SRM were more stable in alkaline and acidic conditions (Fig. 2e,f and Table 1). The stabilization of the photocycle kinetics of sensory rhodopsins was shown to be a hallmark of interactions with cognate transducers^30^. The photocycle kinetics, estimated by the Tau value, of SRM-HtrM were more stable (tau = 4–5 seconds) in both alkaline and acidic pH conditions, while the kinetics of SRM alone were greatly affected by pH (tau = 16.11 and 2.7 seconds in alkaline and acidic conditions, respectively) (Table 1). We consistently observed a similar stabilization of the photocycle kinetics with the HmSRI-HtrI fusion protein (Table 1). Contrary to the extremely slow kinetics of HmSRI alone, which could not be determined, HmHtrI effectively maintained the similar kinetic among alkaline and acidic conditions. Together, these three independent assays demonstrated the interaction between SRM and HtrM.

**Table 1:** Photocycle kinetics (Tau values) of detergent-solubilized sensory rhodopsins with or without cognate transducers in alkaline versus acidic conditions.

pH	HmSRI	HmSRI-HtrI	HmSRM	HmSRM-HtrM
Alkaline^a^	n.d.^b^	4.49 s	16.11 s	5.62 s
Acidic^a^	4.99 s	2.38 s	2.7 s	4.05 s

^a^The alkaline and acidic pH ranges were 7.6–7.9 and 6.1–6.5, respectively.

^b^n.d., not determined (kinetics too slow to be measured).

The Tau value is defined as the time constant corresponding to half of the maximum value in a one-phase decay plot.

### Microscopy-based Phototactic Measurement

**Figure 3:**
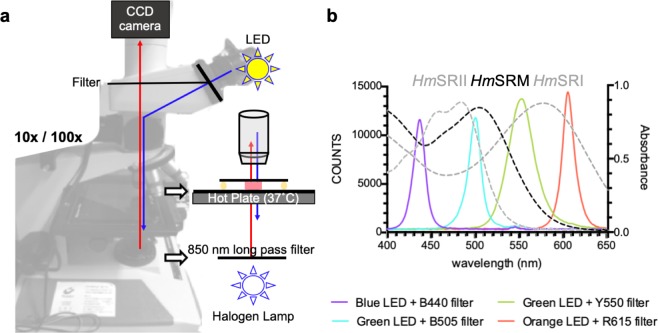
Setup of phototactic measurement system. (**a**) Infrared light (>850 nm) was used as the monitor light for video recording. Cell samples were placed on a thermostatic heat plate (37 °C). To trigger a phototactic response, filtered LED lights were introduced through eyepieces to the samples containing motile cells. (**b**) Spectra of the four LED light sources with peak wavelengths at 437, 501, 554 and 605 nm, each coupled with a corresponding filter, that were used to trigger phototaxis. Relative intensity (photon counts) is shown on the left Y axis. The absorbance spectra of the purified sensory rhodopsins (dashed lines, right Y axis) from *H. marismortui* are plotted to show the overlap with LED lights.

**Figure 4:**
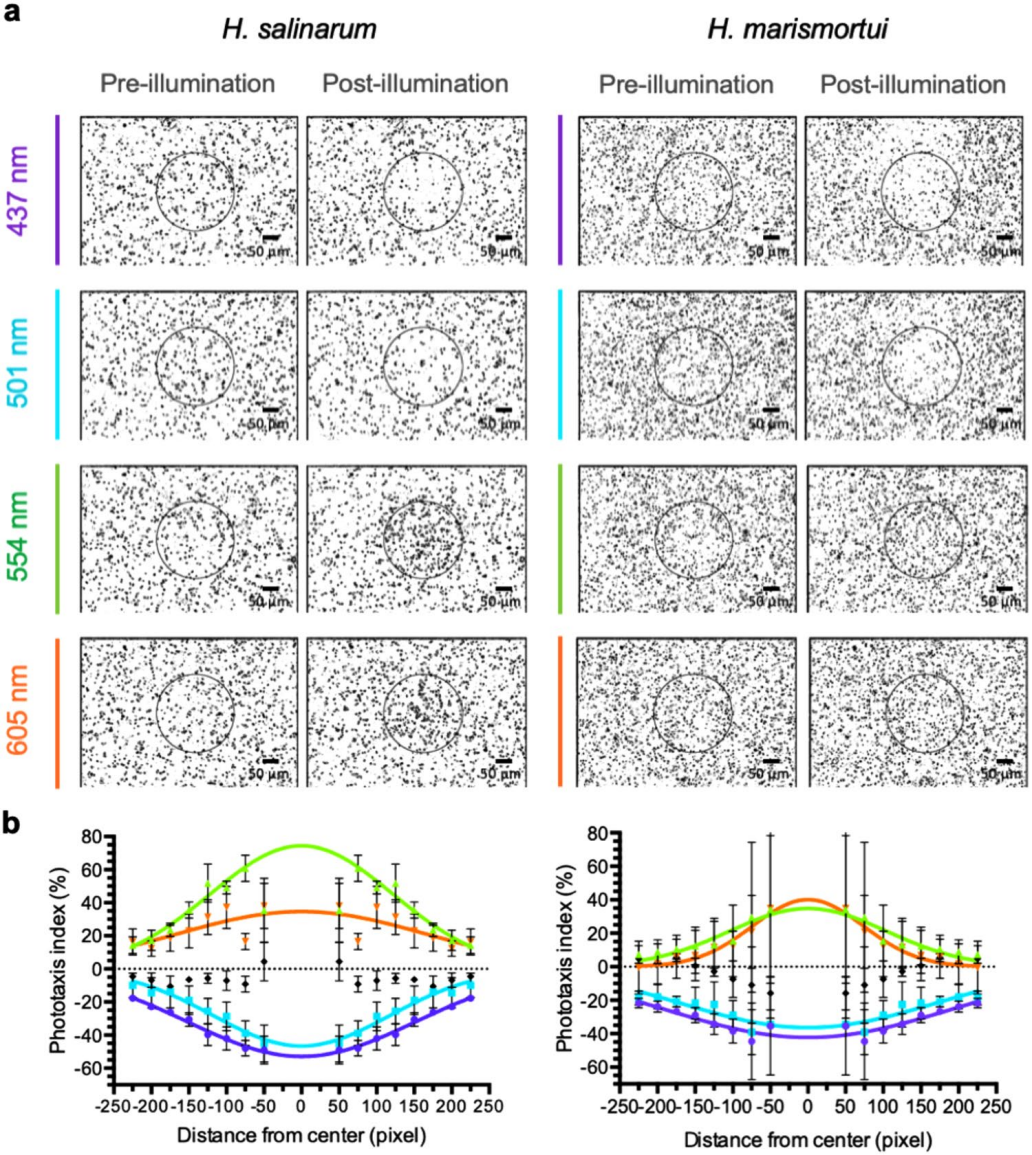
Phototactic responses of *H. salinarum* and *H. marismortui*. (**a**) Microscopy images of motile *H. salinarum* and *H. marismortui* cells before and after 6 minutes of illumination with 437, 501, 554 and 605 nm light. A circle of radius = 125 pixels (drawn in the centre) was used to indicate the central region of the images. (**b**) Phototactic indices at different wavelengths. Curve colours correspond to those wavelengths shown in (**a**), with black indicating the dark control. Error bars indicate the standard deviation of at least 3 biological replicates.

The interaction between SRM and HtrM suggested a functional photosensory signalling system; however, the cytoplasmic side of HtrM lacks the histidine kinase domain required for signal relay to flagellar motors. Nonetheless, HtrM contains a cytoplasmic HAMP domain, which is known to be involved in signal relay from transmembrane receptors^31^. This led us to ask whether the primary function of SRM-HtrM was to modulate the existing phototactic signalling of SRI and SRII. To investigate the potential phototaxis-modulating effect of SRM, we set up a microscopy system (illustrated in Fig. 3a) to monitor the long-term phototaxis of both *H. salinarum* and *H. marismortui*. Four separate LED light sources, each chromatically filtered to ensure wavelength specificity (Fig. 3b), were used to illuminate motile cells through eyepiece to the 100x objective lens (Fig. 4a). The phototactic response was further quantified with a phototactic index by calculation of the cell density before and after the defined circular areas in the field of 10x objective (detailed in Methods). Since the illumination areas were not clearly defined, we assessed the phototactic index at different radii to quantify the phototactic response as shown in Table SI. After illumination for 6 minutes, low and high cell densities within the illuminated region indicate negative and positive phototaxis, respectively.

Using our phototaxis quantification system, we compared cell densities of *H. salinarum* and *H. marismortui* after illumination (Fig. 4). In *H. salinarum*, both negative and positive phototaxis were more dramatic, with −50.0% and +70.0% changes in the phototactic indices after illumination with 437 nm and 554 nm light, respectively (Fig. 4b). Contrary to *H. salinarum*, the phototactic responses of *H. marismortui* were lower, with −40.0% and +40.0% changes in the phototactic indices after illumination with 437 nm and 554 nm light, respectively (Fig. 4b). In particular, the phototactic responses of *H. salinarum* and *H. marismortui* were substantially different at 554 nm, which falls between the maximum absorbances of SRM and SRI. These results showed that the phototactic response of *H. marismortui* was less sensitive than that of *H. salinarum*.

### Transformation of SRM and SRM-HtrM into *H. salinarum* cells

**Figure 5:**
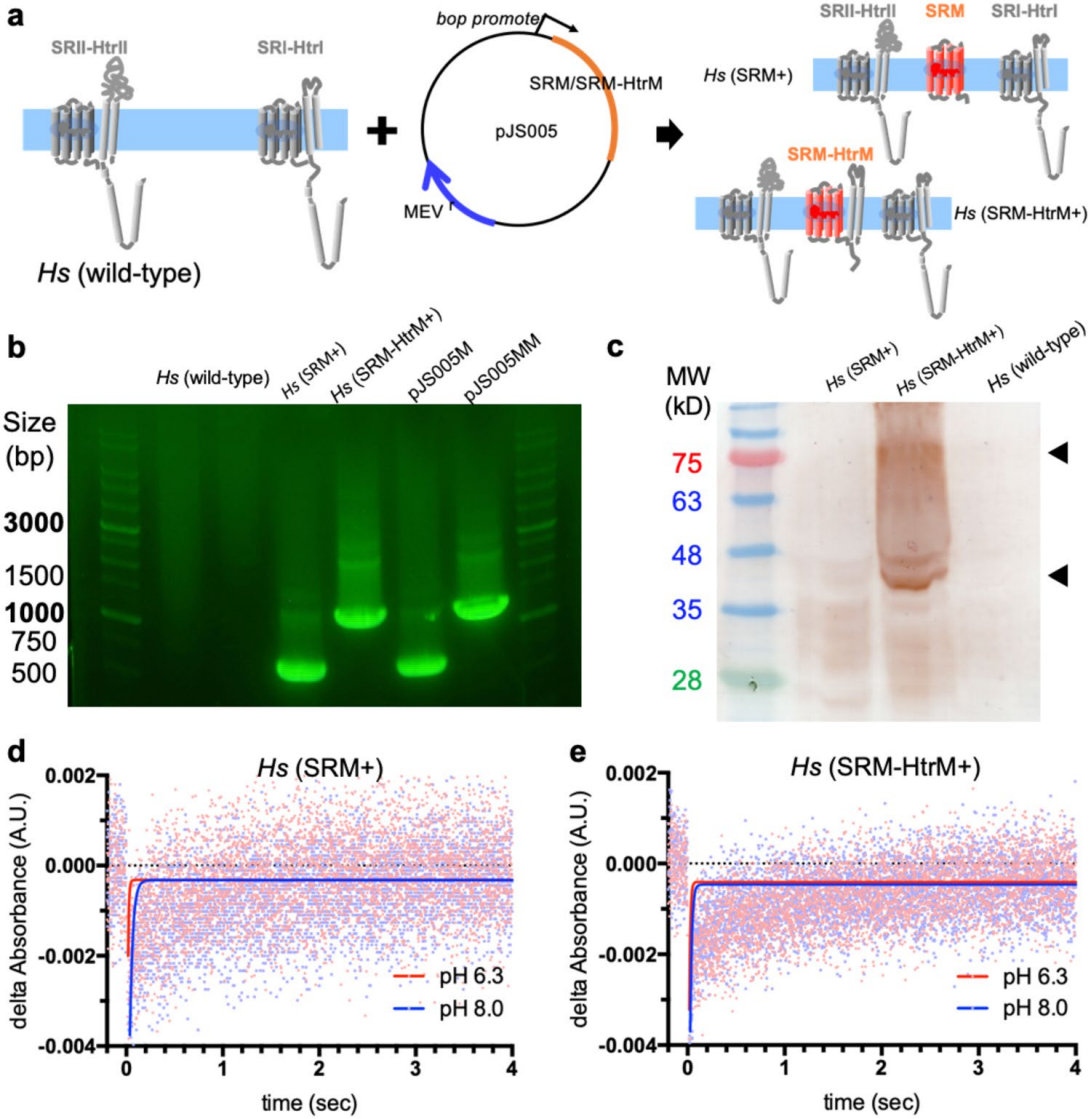
Examination of SRM- and SRM-HtrM-transformed *H. salinarum*. (**a**) Schematic of the SRI/SRII system in *H. salinarum* and the transformed plasmid encoding the SRM or SRM-HtrM genes. The right panel shows the transformed *H. salinarum* cells expressing either the SRM or SRM-HtrM proteins. Transformants were examined by colony PCR (**b**) and western blot (**c**). The size of SRM and SRM-HtrM PCR product is 696 bp and 1300 bp, respectively. The molecular weight of SRM and SRM-HtrM is 23.2 kDa and 42.6 kDa, respectively. (**d, e**) Photocycle measurements of SRM (**d**) and SRM-HtrM (**e**) extracted from the *H. salinarum* membrane. Raw images of blots/gels are presented in Fig. S2.

The low photosensitivity of *H. marismortui* led us to hypothesize that SRM attenuates both positive and negative phototaxis in the blue-green to yellow spectra. To examine this attenuation effect of SRM, we transformed SRM or SRM-HtrM fusion complex into *H. salinarum*, which contains SRI and SRII, and measured phototaxis. Two plasmids, pJS005M and pJS005MM, encoding SRM alone or SRM-HtrM fusion proteins, respectively, were constructed (Fig. 5a). Both plasmids were then transformed separately into *H. salinarum* cells and selected for with mevinolin. Successful transformation was confirmed by the colony PCR (Fig. 5b). The vector (i.e. pJS005M or pJS005MM) was served as the positive control, and the host cell colony (i.e. *Hs* (wild type)) was the negative control. Results showed that the transformant cells contained the amplicon of the proper size as that of the positive control.

The protein expression levels of SRM alone or SRM-HtrM complex in transformed *H. salinarum* were further confirmed by western blot. The expression level of SRM-HtrM complex appeared to be higher than that of SRM alone, indicating that HtrM stabilized SRM in the *H. salinarum* cell membrane (Fig. 5c). Both monomers and dimers of the SRM-HtrM complex could be detected (Fig. 5c, black arrow).

To further examine whether the interaction and stability of the SRM-HtrM fusion protein were intact in the *H. salinarum* cell membrane, photocycle measurements under acidic and alkaline conditions were conducted. The cell membrane was prepared directly from *H. salinarum* transformants, *Hs* (SRM+) and *Hs* (SRM-HtrM+). Under both acidic and alkaline conditions, the photocycle kinetics (Fig. 5d,e) of both SRM and SRM-HtrM were faster in the *H. salinarum* cell membrane than what we observed in detergent (Fig. 2e,f), suggesting that SRM and SRM-HtrM were functioning properly in the *H. salinarum* membrane. The stabilization of the SRM photocycle by HtrM under both acidic and alkaline conditions was similar to those observed in Fig. 2e,f, in which SRM was solubilized in detergent.

### SRM-HtrM attenuated haloarchaeal phototaxis

**Figure 6:**
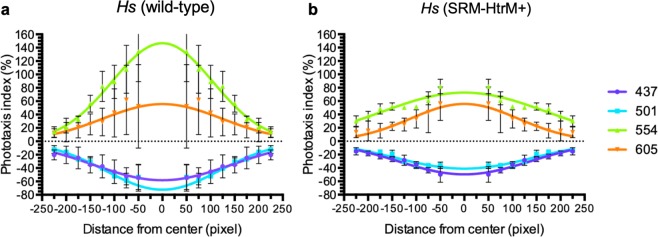
Analyses of *Hs* (wild-type) and *Hs* (SRM-HtrM+) phototaxis. Phototactic indices of *Hs* (wild-type) (**a**) and *Hs* (SRM-HtrM+) (**b**) calculated within circles increasing by increments of 25 pixels (radii = 50, 75, 125, 150, 175, 200, 225 pixels). Illumination conditions correspond to maximum wavelengths shown in Fig. 3b. Exact phototactic index values can be found in Table SI.

To evaluate whether SRM modulates the phototactic signalling of SRI or SRII in *H. marismortui*, a SRM (*xop2*) knock-out is required. However, due to the lack of genome-editing tools that are feasible for *H. marismortui*, we chose *H. salinarum* as a cellular platform to investigate the phototaxis-modulating function of SRM. We transformed the SRM-HtrM fusion protein into *H. salinarum* and measured the phototactic responses of SRI and SRII in the presence of SRM-HtrM fusion protein (Fig. 6). Our results showed that the *Hs* (SRM-HtrM+) transformants were less sensitive to the light which triggered positive and negative phototaxis. Moreover, the attenuation of phototaxis was more obvious in the 501 to 554 nm range (Fig. 6), which corresponds to the absorbance of SRM-HtrM (Fig. 3b). The attenuated phototaxis indices of *H. salinarum* in the presence of SRM-HtrM (Fig. 6, right panel) was close to the phototaxis of *H. marismortui* (Fig. 4b, right panel). In conclusion, our results showed that SRM-HtrM from *H. marismortui* attenuated phototactic responses to 501 nm and 554 nm illumination mediated by SRI and SRII in *H. salinarum*.

## Discussion

Organisms on Earth have developed various photoreceptors to capture solar energy and regulate physiological responses. The maximum number of microbial rhodopsins in a single haloarchaeon was four until we reported a six microbial rhodopsin system in *Haloarcula marismortui*^24^. Those six microbial rhodopsin proteins include the unique and functionally unknown SRM-HtrM. Such an extraordinary number of microbial rhodopsin proteins suggests a more flexible and able survival strategy. In previous studies we identified a dual bacteriorhodopsin configuration, which was never before observed, composed of two BRs with different optimal pH tolerances^32^ for carrying out their functions. These two BRs work together to enhance the ability of *H. marismortui* cells to raise the proton motive force^33^ through their light-driven outward proton pumping, which facilitates proton influx from the extracellular side through ATP synthase for more efficient energy production.

We previously showed that the three-sensory rhodopsin system in *H. marismortui* is constitutively expressed^24^. The downstream phototactic signalling mechanism in *H. marismortui* is not well known. Nonetheless, from previous studies it is widely accepted that cognate transducers of sensory rhodopsins interact with chemotaxis components to regulate the flagella motor^13,15,16,34^. Therefore, we speculated that the light-activated SRM-HtrM complex transmits a “null” phototactic signal in *H. marismortui* through the sole cytoplasmic HAMP domain of HtrM, such that both positive and negative phototaxis would have been attenuated. In particular, HtrM may compete for the binding of certain chemotaxis components, such as CheB or CheR, and decreases their availability for binding of either HtrI or HtrII. The effect of SRM-HtrM on SRI and SRII shown in this study suggests that *H. marismortui* has an RGB-like sensory system. Further studies are required to elucidate the signalling mechanism by which SRM-HtrM attenuates phototactic responses mediated by SRI and SRII.

The effect of SRM-HtrM on SRI and SRII shown in this study suggests an RGB-like sensory system for *H. marismortui*. The phototaxis results observed in the wild type and SRM-HtrM expressing *H. salinarum* cells provided us some clues for further phototaxis research. We applied four specific wavelengths of light ranging from the violet to the red visible light spectra. SRI- and SRII-mediated phototactic effects peaked at 605 and 437 nm, respectively. On the other hand, in the group illuminated with wavelengths between 437 nm and 501 nm, negative phototaxis was inhibited in the *Hs* (SRM-HtrM+) cells. Comparing the 554 nm and 605 nm groups, the relatively elevated positive phototaxis in 554 nm group was diminished in *Hs* (SRM-HtrM+). These results showed that SRM-HtrM attenuates both the SRI and SRII systems at an optimal wavelength range of 520–550 nm. HtrM is therefore capable of affecting both positive and negative phototaxis with its sole HAMP domain.

From a technical standpoint, this study established a robust phototactic quantification system that can be implemented with conventional optical microscopes (100x objective) with ease. The system captures long-term, population-level behaviour of motile *H. salinarum* and *H. marismortui* cells. Given the small cell size (1–2 µm)^35^ and slow motility (approximately 2.3 µm/s)^10^ of *H. marismortui*, the system can be readily applied to other microbial species with larger cell bodies and faster motility. In addition, methods of image processing used in our assay are straightforward and can be implemented with ImageJ.

It is worth mentioning that the transformation or “transplantation” of SRM-HtrM fusion complex into *H. salinarum* is an alternative strategy to bypass the gene knockout approach in haloarchaea. Different from *H. marismortui*, *H. salinarum* cells have a dual-sensory rhodopsin system that includes positive (SRI) and negative (SRII) phototactic sensors. We cloned a peptide-linked SRM-HtrM complex into the open reading frame under the regulation of the *bop* promoter, which is known to be turned on in harsh environments such as low nutrients, low oxygen and intense illumination^36^. We performed PEG600-assisted transformation on *H. salinarum* and obtained *Hs* (SRM-HtrM+) transformants. The proper functioning of the transformed SRM-HtrM complex in the *H. salinarum* lipid membrane was supported by the finding that HtrM stabilized the photocycle of SRM under different pH conditions.

We also concluded that SRM-HtrM is a new type of sensory rhodopsin based on its biophysical and biochemical properties. The following points support the designation of SRM as a sensory rhodopsin: (1) SRM shares 63.2% identity with HmSRI, 64.0% identity with HsSRI, 66.2% identity with HmSRII, and 67.8% identity with HsSRII. (2) The maximum absorbance is different from any sensory rhodopsin in *H. marismortui*. The maximum absorbance of SRM is 504 nm, which differs from the maximum absorbances of SRII at 485 nm and SRI at 585 nm. (3) SRM shares the slow photocycle kinetics observed for other known sensory rhodopsin proteins. The slow photocycle rate is essential for accumulation of M and/or O intermediate state, in which sensory rhodopsin can activate phosphorylation cascade^37^. The photocycle kinetics of SRM-HtrM occur in 5 seconds, the same as what has been observed for SRI and SRII and unlike what has been recorded for BR or HR, which take only milliseconds to complete a photocycle. (4) SRM possesses a cognate transducer, HtrM. The interaction between SRM and HtrM was shown by several assays. The photocycle of SRM oscillated dramatically between different pH conditions and salinities without the binding of HtrM, while it was stable for the SRM-HtrM complex. This transducer complex-induced stabilization is consistent with previous studies of SRI and SRII and their corresponding cognate transducers. In addition, the unique structure of the partner cognate transducer, HtrM, is not observed in any other haloarchaea. In terms of phototactic signalling, HtrM lacks the methyl-accepting domain and the tip region for CheA/CheW complex docking. Instead of two HAMP domains, which have been identified in both HtrI and HtrII, HtrM only has one HAMP domain immediately following the two transmembrane domains. The interaction of CheA/CheW with HtrM is therefore unlikely to occur upon light-activation. (5) Most importantly, SRM attenuates the phototactic responses mediated by SRI and SRII.

In this study, we successfully generated an *H. salinarum* transformant, which can serve as a valid control for future signalling pathway studies in *H. marismortui*. We report the physiological function of unique SRM-HtrM system previously characterized in *H. marismortui* by our group^24^. SRM-HtrM was successfully transformed into the two sensory rhodopsin-containing *H. salinarum* and the transformants showed attenuated HsSRI and HsSRII-mediated phototactic responses to green light. The optimal wavelength range for SRM-HtrM function was 520–550 nm. Our results suggest that the SRM-HtrM complex is therefore a new type of sensory rhodopsin and this cognate transducer signalling complex modulates the sensitivity of haloarchaea phototactic responses. Such unique three-sensory rhodopsin system indeed work coordinately. Together with the dual-BR system we previously reported, they both contribute to the maximal survival of *H. marismortui* cells in resource-limited Dead Sea.

## Methods

### Plasmid Construction

The *xop2* (HmSRM) and *xhtr2* (HmHtrM) genes were cloned from *H. marismortui* genomic DNA. All plasmids were prepared and maintained in *Escherichia coli* strain DH5α. For protein expression, the *xop2* gene with a C-terminal hexa-histidine tag (6xHis-tag) was cloned into pET-21b with *Nde*I/*Hin*dIII restriction sites. The *xop2-xhtr2* fusion protein was constructed as described in previous studies^30,38,39^ with a flexible-linker (-ASASNGASAH) between *xop2* and *xhtr2* and a C-terminal hexa-His tag.

For *H. salinarum* transformation, the *xop2* gene with the C-terminal 6xHis-tag was inserted into the pJS005 vector (which contains a *bop* promoter to overexpress the target protein) with *Nco*I/*Xba*I restriction sites. The *xop2-xhtr2* fusion protein was inserted into the pJS005 vector similarly, but with *Nco*I/*Xho*I restriction sites, a flexible linker (ASASNGASAH) between *xop2* and *xhtr2*, and a 6xHis-tag at the C-terminus of *xhtr2*. The primer sequences used for the cloning of *xop2* were F: 5′-ggatccccatggcacaagagatcgtttggtac and R: 5′-ctgccatatgcgccgacgcgccgttcgacgccgacgc-cttggcgggagctacggacc, and for *xhtr2* were F: 5′-gatatacatatgtcggcagtaaccaagcgg and R: 5′-cgactctagaaagcttctaatggtgatggtgatggtgcgctgctcgggaatcgatctctgcgtc. The underlined sequences indicate the intrinsic *xop2* or *xhtr2* gene sequences. The sequences of all plasmid constructs were verified by Genomics Co., Ltd, Taipei, Taiwan.

### Protein Expression and Purification

Unless otherwise indicated, all liquid cultures were grown in LB at 37 °C with shaking at 150 rpm. The pET-21b plasmids containing *xop2* and *xop2*-*xhtr2* were transformed into *E. coli* strain C43(DE3) available from Lucigen® Overexpress™ competent cell. A 3 ml overnight culture was grown from a single colony and then subcultured in 16 ml before inoculation into 800 ml in a 2-liter Erlenmeyer flask. Protein expression was induced during exponential phase (OD_600_ = 0.4–0.6) with 1 mM IPTG and 10 nM methanol-solubilized all-trans retinal. Cells were harvested after 4 hours of induction by centrifugation at 6,000 xg for 10 min (4 °C). Cell pellets were resuspended with ice-cold lysis buffer (4 M NaCl, 50 mM Tris-HCl, pH 7.8), followed by the addition of 200 μM PMSF and 14.3 mM 2-mercaptoethanol. Cells were lysed by sonication (Misonix® Sonicator 3000) with an energy of 69 W, with 5/5 seconds of on/off cycles and a total approximately 5 minutes of “on” time. Large cell debris were pelleted and discarded by centrifuging at 16,000 xg for 10 min at 4 °C. From the supernatant, the membrane protein fraction was further pelleted and collected by ultracentrifugation at 100,000 xg for 1 h, at 4 °C. The membrane protein fraction pellets were resuspended in ice-cold lysis buffer supplemented with 2% n-Dodecyl β-D-maltoside (2%-DDM lysis buffer) and rotated at 20 rpm for 16 h at 4 °C to solubilize the membrane proteins. Unsolubilized debris were pelleted and discarded by centrifuging at 36,000 xg for 45 min at 4 °C. Solubilized proteins were bound to a Ni Sepharose High Performance column (GE Healthcare) by rotating at 20 rpm for 5 h, at 4 °C. Finally, proteins were washed and eluted with 0.02%-DDM lysis buffer containing 20, 50, and 250 mM imidazole and dialyzed to the specific pH and salt conditions indicated in Fig. 2.

### Protein Stability Test

Purified proteins were freshly dialyzed to specific conditions (as indicated in Fig. 2) at 4 °C with a 6–8 kDa cut-off dialysis membrane and stirred at 40–50 rpm overnight. Before UV-Vis spectrum measurements, precipitates were removed by centrifuging at 13,000 xg for 10 min at 4 °C. Spectra were normalized by absorbance at 280 nm to compare protein stability.

### *H. salinarum* Transformation

A single colony of *H. salinarum* NRC-1^40^ was inoculated into 2 ml of CM+ medium^41^ to stationary phase. The cells were then subcultured (20 µl culture into 2 ml CM+ medium) at 42 °C with 180 rpm shaking under constant white LED illumination to an OD_600_ = 0.4–0.6, which took 36 hours. The cultures were then subcultured again (150 μl of the culture into 15 ml CM+ medium) under the same conditions to an OD_600_ = 0.4–0.5. A total of 2 ml of the culture was then harvested by centrifugation in a 15-ml Falcon tube at 750 × g for 15 min at room temperature. The cell pellets were resuspended in 200 µl of spheroplast solution (SPS; 2 M NaCl, 15% sucrose, 27 mM KCl, 50 mM Tris base, pH 8.5). The cell suspension was directly mixed with 10 µl of 0.5 M EDTA, immediately followed by 30 µl of plasmid DNA solution. After incubating for 5–10 minutes at room temperature, 240 µl of 50% PEG_600_ SPS was slowly added and the solutions were mixed softly by inverting the tubes 20–30 times, followed by another 30 min incubation. The solution was then washed twice with 5 mL of CM+ sucrose medium and centrifuged at 750 × g for 15 min at room temperature. The cells were then recovered at 37 °C for 24 h with shaking at 130 rpm under constant white LED light. Finally, 50 µl of the recovered culture was spread onto an SRMEV plate (20 g MaSO_4_x7H_2_O, 3 g sodium citrate, 2 g KCl, 250 g NaCl, 3 g yeast extract, 5 g tryptone, 15 g agar in 1-liter dH_2_O, pH 7.0, supplemented with 40 µg/ml mevinolin), from which candidate colonies were picked after approximately two weeks.

The positive transformants were confirmed by colony PCR using *xop2_f* (5′-attcggatccatggcacaagagatcgtttggtac) and *xop2_r* (5′-ggccgcaagcttcttggcgggagctacgga-cc) primers for *H. salinarum* (SRM+), and *xop2_f* and *xhtr2_r* (5′-ggccgcaagctttcggga-atcgatctctgcgtc) primers for *H. salinarum* (SRM-HtrM+). Protein expression analysis in the positive transformant was performed by growing at 37 °C with shaking at 180 rpm to an OD_600_ = 0.8–0.9 in a glass tube with a 1:1 liquid:air ratio. A total of 1.5 ml of culture was harvested by centrifuging at 12,000 xg at room temperature for 30 seconds. The cell pellet was then lysed with 100 µl of distilled water. The protein expression level was verified by Western blot (QIAGEN® Penta-His HRP conjugate kit) after sonicating the resuspended cells using a microtip on ice (6 W, 1 sec “on”, 1 sec “off”, total in 10 sec).

### *H. salinarum* Membrane Extraction

Wild-type and transformed *H. salinarum* were cultured from a single colony in 3 ml of halomedium^42^. After culturing at 37 °C with shaking at 180 rpm for 3 days, 500 µl was subcultured into 16 ml of halomedium for another 3 days to stationary phase, which served as the seed culture. A total of 16 ml of the seed culture was then added to 800 ml of halomedium and shaken under constant white LED illumination until late exponential phase (OD_600_ = 0.8–1.2), in which the proteins were induced by the white light in a condition of oxygen-deficiency during stationary-phase. Cells were pelleted by centrifugation at 6000 xg for 10 min at 4 °C and then resuspended in 30 ml of cold lysis buffer (4 M NaCl, 50 mM MES, pH 5.8). To fragment the cell membrane, cells were sonicated at 69 W with 5/5 sec on/off cycles and a total on” time of 5 min. After sonication, cell debris were removed by centrifuging at 36,000 xg for 45 min at 4 °C, and then the membrane fraction was pelleted at 100,000 xg for 1 h, at 4 °C. Finally, the membrane fraction was resuspended in 20–30 ml of cold lysis buffer. The pH of the different samples was adjusted by adding 10X lysis buffers with specific pH values ranging from 5.8 to 8.0.

### Photocycle Measurement

The photocycle measurement system was first proposed by Goldschmidt et al, and pratically described by Fu *et al*.^24,43^ In brief, protein samples were loaded into a quartz cuvette with three side windows, two of which were used to monitor the light that measures the absorbance of the sensory rhodopsins at specific wavelengths. The third window is used for the pulse laser (LS-2137U-N, LOTIS LII®, Japan) that triggers the photocycle. To monitor the absorption change of protein samples, the white light (SL1 Tungsten Halogen Light Source, StellarNet Inc., USA) was directed through an optical fibre (1 mm in diameter) to pass through the sample in the quartz cuvette, then filtered by the monochromator (DMC1–02, Optometrics®, USA) at 503 nm, finally reaching a photomultiplier tube (E717, Hamamatsu®, Japan). The signal from the photomultiplier was displayed on an oscilloscope (DPO4032, Tektronix®, USA). To trigger the photocycle, a pulse of Nd:YAG laser (20 W, 532 nm, 2 ns duration) was directed into the cuvette perpendicular to the light path of the monitoring light. Time-series results were averaged from 8 data sets.

### Phototaxis Indexing of Haloarchaea

Motile cells were selected at least twice on halomedium plates with 0.3% agar. A 20 µl aliquot of agar on the outer edge of the cell mass was inoculated into 10 ml of halomedium in a glass tube (air:liquid = 1:1). For *H. salinarum*, the culturing conditions were 37 °C with shaking at 180 rpm to an OD_600_ = 0.8–1.0. The culture was then incubated without shaking at 37 °C overnight to sediment the debris and dead non-motile cells. A total of 10 µl of the surface of the liquid culture was diluted into 40 µl of halomedium without peptone. For *H. marismortui*, the culturing conditions were 37 °C with shaking at 180 rpm to an OD_600_ = 1.40, followed by immediate dilution into halomedium (1:3) before measuring phototaxis. A total of 0.5 µl of diluted motile-cell culture was applied to a Vaseline-coated glass cover slip. This glass cover slip was then assembled with a glass sample slide pre-treated with two separated strips of cellulose tape on two sides to serve as a spacer between the cover slip and sample slide. The sample in this cassette had a thickness of approximately 30–50 µm (calculated from the sample volume divided by the area after cassetting). Samples were then secured on a microscope (Olympus BH-2) with a thermostatic plate set at 37 °C. Long passed 850 nm infrared light was used as the monitor light to take micrographs with a 10x objective and a greyscale CCD camera (MTV-62V6HN, MINTRON®, New Taipei City, Taiwan) (Fig. 3a). All filters for the phototaxis triggering light were manufactured by Rocoes® Electro-Optics Co., Ltd. Filtered LED (DL®, DLSL-5W) light (spectrums described in Fig. 3b) was introduced to the samples via the eyepiece and a 100x objective for 6 minutes (Fig. S1). Photographs taken under the 10x objective before and after light illumination were analysed by ImageJ^44^ (Fig. 4a, Table S2). The ratio of cell counts inside (Cin) the analysis circles to the cell counts out of the defined area (Cout) was calculated. Phototactic indices (%) were calculated by the following equation: [(Cin/Cout)t6 - (Cin/Cout)t0]/(Cin/Cout)t0 * 100%. Positive index values indicate positive phototaxis, while negative values indicate negative phototaxis.

## Supplementary information

Supplementary Information

## Data Availability

The data generated and analysed in this study are available from the corresponding author on reasonable request.
