# Supplementary material for: The Blue-Green Sensory Rhodopsin SRM from Haloarcula marismortui Attenuates Both Phototactic Responses Mediated by Sensory Rhodopsin I and II in Halobacterium salinarum
Source: Sci Rep. 2019 Apr 5;9:5672. doi: 10.1038/s41598-019-42193-y (PMC6450946; doi:10.1038/s41598-019-42193-y)
Supplement: Supplementary file 1 — Supplementary Information [file 41598_2019_42193_MOESM1_ESM.pdf]

The Blue-Green Sensory Rhodopsin SRM from *Haloarcula marismortui* Attenuates Both Phototactic Responses Mediated by Sensory Rhodopsin I and II in *Halobacterium salinarum*

Jheng-Liang Chen[1], Yu-Cheng Lin[1], Hsu-Yuan Fu[1], and Chii-Shen Yang[1,*]

[1]Department of Biochemical Science and Technology, National Taiwan University, Taipei, 10616, Taiwan

[*]Chii-Shen Yang: chiishen@ntu.edu.tw

Supplementary Information

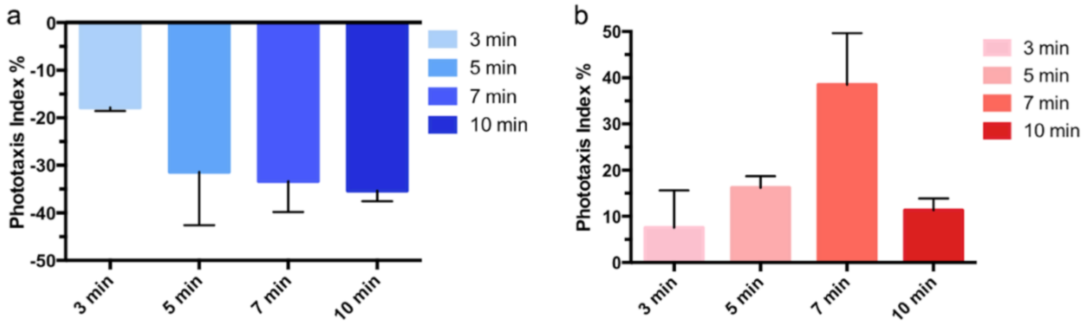

Figure S1. Response time of positive and negative phototaxis. Blue and orange LED light were introduced to illuminate the cell samples and induce (a) negative and (b) positive phototaxis, respectively. Illumination times of 3, 5, 7 and 10 minutes are shown here. Negative phototaxis shows a saturated result with illumination above 5 minutes. Positive phototaxis shows an optimal result with illumination between 5 and 7 minutes. The data presented here was analysed from a circle of radius = 335 pixel, with a sample number $N = 3$.

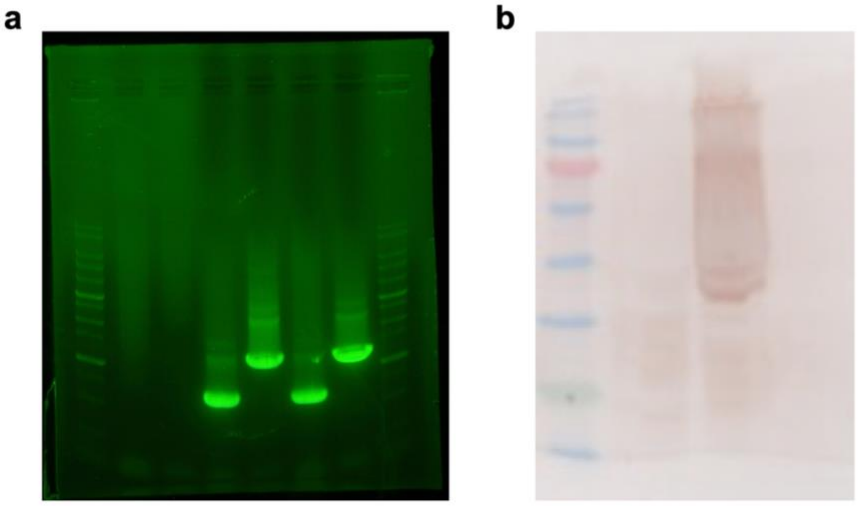

Figure S2. Full-length (a) colony PCR gel and (b) Western blot of *H. salinarum* transformant.

Table SI. Average phototactic index % of each analysed circle and its standard deviation (SD) and sample number. R: the radius of the defined analysed circle. M: the mean of the phototactic index of each data set. S: the standard deviation of each data set. *N*: the sample number of each data set. The purple, cyan, green and orange columns represent the illumination conditions of the spectrum peaks at 437 nm, 501 nm, 554 nm and 605 nm, respectively.

	Hs (wild-type)			*Hs* (SRM-HtrM+)				*Hs* (wild-type)			*Hs* (SRM-HtrM+)		
R	M	S	*N*	M	S	*N*	R	M	S	*N*	M	S	*N*
50	-54.52	20.10	4	-49.72	11.73	3	50	134.30	44.93	3	81.90	10.78	5
75	-52.21	12.85	4	-43.51	4.02	3	75	107.63	35.80	3	60.81	6.57	5
100	-45.54	17.56	4	-35.01	4.65	3	100	88.84	24.96	3	51.66	2.29	5
125	-37.56	17.22	4	-28.43	7.6	3	125	82.09	22.55	3	50.45	2.85	5
150	-35.11	12.23	4	-26.75	3.90	3	150	56.22	12.61	3	51.97	5.53	5
175	-24.83	16.35	4	-22.97	3.77	3	175	32.77	5.09	3	48.24	7.58	5
200	-20.33	13.12	4	-18.33	3.63	3	200	20.37	7.92	3	37.41	7.23	5
225	-20.23	7.41	4	-15.06	5.47	3	225	13.85	7.83	3	30.05	7.93	5
50	-53.76	18.45	3	-36.11	4.85	3	50	52.21	62.40	3	56.12	25.12	3
75	-58.75	10.97	3	-36.00	3.38	3	75	61.87	51.75	3	43.29	30.64	3
100	-52.07	2.54	3	-36.82	4.06	3	100	42.59	36.15	3	33.21	13.61	3
125	-40.52	2.42	3	-30.22	1.52	3	125	28.85	20.69	3	24.96	9.63	3
150	-30.27	5.51	3	-18.98	5.40	3	150	25.29	15.61	3	22.31	6.25	3
175	-26.74	2.22	3	-15.84	3.44	3	175	21.27	16.23	3	15.60	11.17	3
200	-16.04	1.41	3	-16.55	2.26	3	200	16.98	17.71	3	15.28	14.77	3
225	-12.34	5.83	3	-14.14	2.99	3	225	12.15	6.72	3	13.21	8.01	3

Table SII. Commands for image analysis in ImageJ2.

To Enter Batch Process Interface
Process > Batch > Macro…

Auto Adjust Brightness and Contrast

```
AUTO_THRESHOLD = 5000;
 getRawStatistics(pixcount);
 limit = pixcount/10;
 threshold = pixcount/AUTO_THRESHOLD;
 nBins = 256;
 getHistogram(values, histA, nBins);
 i = -1;
 found = false;
 do {
         counts = histA[++i];
         if (counts > limit) counts = 0;
         found = counts > threshold;
 }while ((!found) && (i < histA.length-1))
 hmin = values[i];

 i = histA.length;
 do {
         counts = histA[--i];
         if (counts > limit) counts = 0;
         found = counts > threshold;
 } while ((!found) && (i > 0))
 hmax = values[i];

 setMinAndMax(hmin, hmax);
 print(hmin, hmax);
```

AutoAdjust Threshold

```
run("8-bit");                          # convert the images to 8-bit
run("Find Edges");                     # enhance the edges of cells in image
setAutoThreshold("Default dark");      #auto adjust the threshold and make images
                                         white and black
```

//run("Threshold...");
 setOption("BlackBackground", false);
 run("Convert to Mask");

Particle (Cell) Counting in Whole Field

makeRectangle(15, 9, 690, 469);
 #To make a rectangle without the frame in each image
 run("Analyze Particles...", "size=18-Infinity show=Outlines summarize");
 # particle size was 18 in *H. salinarum* and 15 in *H. marismortui*

Particle (Cell) Counting in Defined Area

makeOval(40, 160, 200, 200);
 #To make an oval with following dimensions (x, y, width, height). For example, to make a circle with radius of 100 pixels in the centre of the image with 720 x 480 pixels
 run("Analyze Particles...", "size=18-Infinity show=Outlines summarize");
 # particle size was 18 in *H. salinarum* and 15 in *H. marismortui*
